# Photoluminescent, “ice-cream cone” like Cu–In–(Zn)–S/ZnS nanoheterostructures

**DOI:** 10.1038/s41598-022-09646-3

**Published:** 2022-04-06

**Authors:** Xue Bai, Finn Purcell-Milton, Daniel K. Kehoe, Yurii K. Gun’ko

**Affiliations:** School of Chemistry and CRANN Institute, Trinity College, University of Dublin, Dublin, D02 Ireland

**Keywords:** Chemistry, Materials chemistry, Optical materials

## Abstract

Copper based ternary and quaternary quantum confined nanostructures have attracted huge attention over recent years due to their potential applications in photonics, photovoltaics, imaging, sensing and other areas. However, anisotropic nanoheterostructures of this type are still poorly explored to date, despite numerous predictions of the distinctive optical properties of these highly fluorescent heavy metal free nanostructures. Here, we report new fluorescent multicomponent Cu–In–(Zn)–S/ZnS nanoheterostructures with a unique anisotropic “ice-cream cone” like morphology. These nanostructures have been prepared with a seeded growth technique and exhibit distinct photophysical properties with maximum emission in the visible range (≈ 640 nm) and long photoluminescence lifetimes (τ_average_ ≥ 300 ns). In depth time interval studies have been carried out to better understand the step by step growth mechanism of this distinct “ice-cream cone” like geometry. We have demonstrated that the crystal structure evolution from the zinc blende Cu–In–S core to the wurtzite “ice cream cone” like Cu–In–(Zn)–S/ZnS nanocrystals plays a key role in the origin of this morphology. This research opens new possibilities to produce unique fluorescent Cu-based multicomponent anisotropic heteronanostructures, while also offering a distinctive insight into the design of bespoke nanostructures, which could find a range of potential applications.

## Introduction

Controlling the morphology of semiconducting nanocrystals (NCs) has always been a very important area since the optical properties of quantum dots (QDs) are strongly dependent on their morphologies^1,2^. Much effort has been focused on the investigation of the growth mechanisms for anisotropic geometries^3–8^ in order to control the synthesis to a finer level, enabling the preparation of anisotropic quantum structures for particular applications^9,10^. Fluorescent Cd-based QDs with nonspherical shapes are widely investigated^3,11–14^ including complex heterostructures such as rod-in-rod structures^15^ or dot-in-rod structures^16^. Previous studies have shown that WZ (Wurtzite) seeds are necessary for the formation of 1D nanorod structures while ZB (zinc blende) seeds are the basis for the preparation of branched nanostructures^17,18^. In contrast, the exploration of Cu-based quantum dot anisotropic nanomaterials has only been developed to a very limited level, with a minimal range of nonspherical shapes being reported including ternary or quaternary nanoplatelets^19–22^, nanorods^23–28^, tetrapods^29^, nanocubes^30^ and core/shell/shell anisotropic nanonail heterostructures that have been developed by our group recently^9^.

Even in these cases, not all of the nanostructures demonstrated sufficient fluorescent properties, which are necessary to take full advantage of these structures. An interesting example of these nanostructures was reported by Ward van der Stam and co-workers, involving NIR emitting CuInSe_2_/CuInS_2_ dot-in-rod structures which were prepared through a complex cation exchange technique^31^. In addition, two groups prepared fluorescent CuInS_2_/CdS heterotetrapod NCs^32,33^, though in both of these examples, the tetrapods still contained the highly toxic element Cd, which hinders their practical applications. Importantly, the growth mechanisms proposed for these two similar tetrapod structures are distinct, with one composed of a CH (chalcopyrite) CuInS_2_ core and WZ CdS arms^32^, while the other involved the growth of WZ CdS arms on a ZB CuInS_2_ core^33^.

The growth mechanisms that have been proposed for Cu-based multicomponent anisotropic nanostructures are consistent with those proposed for much more comprehensively studied Cd-based quantum nanostructures currently. The above case is an exception (both ZB and CH seeds can be used to prepare tetrapod structure), which indicates that the growth mechanisms for Cu-based multicomponent anisotropic geometries have not yet been fully clearly understood. Therefore, there is an urgent need to further explore the synthesis and properties of these anisotropic Cu-based multicomponent fluorescent NCs which are currently of high interest due to their unique optical characteristics and broad range of potential applications^34–38^.

In order to realize the optimal optical properties of most QDs, core–shell QDs are commonly formed, especially structures with a type I band alignment, for example, when a CdS or ZnS shell can be coated onto QD seeds^39–41^. The wide band gap (3.7 eV)^42^, low toxicity and small lattice spacing mismatch between ZnS and the multicomponent Cu-based core (ternary or quaternary)^43^ renders it the best option to passivate the bare Cu-based core and therefore produces a NC with both higher photoluminescence quantum yield (PLQY) and photostability^34,44–49^. For example, Zhang et al. have previously reported that after the ZnS shell coating, the PLQY of the Cu–In–S QDs has significantly increased to over 80%, which is among the highest values ever reported for this type of QDs^44^.

The two-step seeded growth technique is one of the most common means to prepare core/shell heterostructures especially for anisotropic morphologies^14,50–53^. It is possible to control the synthesis via tuning the addition rate of the shell precursors and quenching the reaction at any time point (controlling the shell thickness grown onto the core), which is especially important for the delicate reactions to obtain Cu-based multicomponent anisotropic heterostructures. Compared to spherical QDs, anisotropic elongated nanostructures have a range of unique physical properties including a high aspect ratio, optical polarization anisotropy, polarized light emission, giant birefringence and other parameters, that makes them more suitable for advanced optoelectronics, energy harvesting and biological imaging applications. However, it is very challenging to prepare luminescent nanoheterostructures with anisotropic elongated morphologies. In this work, fluorescent Cu-based multicomponent Cu–In–(Zn)–S/ZnS anisotropic “ice-cream cone” like nanoheterostructures have been prepared using the seeded growth approach. Structures and optical properties of the new nanoheterostructures have been investigated. In addition, the mechanism of growth and formation of these unique nanostructures have been studied in detail. We believe that our preparation methodology could be applied for development of a range of other unique nanoheterostructures with interesting morphologies and properties.

## Experimental section

### Materials

All the reagents were used as received without further purification. Copper iodide (CuI, 99.99%), indium acetate (In(Ac)_3_, 99.99%), 1-dodecanethiol (1-DDT, ≥ 98%), 1-octadecene (ODE, ≥ 95%), zinc stearate (Zn(St)_2_, technical grade), oleic acid (OA, ≥ 99%), oleylamine (OAm, ≥ 98%) have been supplied by Sigma-Aldrich.

### Synthesis of Cu–In–S QDs

Cu–In–S seeds were prepared according to a previously reported procedure with minor modifications^33^. Briefly, 0.0952 g (0.5 mmol) of CuI, 0.146 g (0.5 mmol) of In(Ac)_3_, 10 mL (8 mmol) of 1-DDT and 10 mL of ODE were mixed in a 100-mL three-neck round-bottom flask. The mixture was degassed at 30 °C for 30 min after which it was heated to 210 °C under argon flux. The reaction temperature was kept at 210 °C for 2 h. Then the reaction vessel was allowed to cool to RT. Ethanol was used to remove excess organic materials and precipitate the QDs by centrifugation. The purified Cu–In–S QDs were stored in toluene.

### Synthesis of Cu–In–(Zn)–S/ZnS “ice-cream” nanoheterostructures

#### Preparation of precursor solution

0.6323 g (1 mmol) of Zn(St)_2_ was dissolved in 2.5 mL of OA, 2.5 mL (2 mmol) of 1-DDT and 5 mL of ODE in a 100-mL three-neck round-bottom flask. The mixture was degassed at RT for 20 min under stirring then the atmosphere was switched to argon.

#### Synthesis of Cu–In–(Zn)–S/ZnS heterostructures

3 mL of Cu–In–S QD solution, 5 mL of OAm and 10 mL of ODE were loaded in a 100-mL three-neck round-bottom flask and degassed at 100 °C for 30 min. Under argon atmosphere, the temperature was increased to 230 °C. When the temperature reached 200 °C, the Zn(St)_2_ precursor solution was injected into the reaction vessel using a syringe pump (KD Scientific, Model No: KDS-100-CE, volume = 10 mL, injection rate = 5 mL/h) with the injection taking 2 h. Aliquots were taken out from the reaction vessel at certain time intervals (30 min, 60 min, 120 min) for further studies. The aliquots were taken from the reaction vessel at these certain time intervals are denoted as Cu–In–(Zn)–S/ZnS-30, Cu–In–(Zn)–S/ZnS-60 and Cu–In–(Zn)–S/ZnS-120, respectively. After the injection was completed, the heating mantle was removed, and the reaction vessel was allowed to cool to RT. Toluene and ethanol were used to clean the sample with centrifugation with the clean sample stored in toluene.

### Characterization

#### Electron microscopy

The Cu–In–S seeds, Cu–In–(Zn)–S/ZnS core/shell structures (after 30 min), Cu–In–(Zn)–S/ZnS core/shell structures (after 60 min) and Cu–In–(Zn)–S/ZnS core/shell structures (after 120 min) were characterized by transmission electron microscopy (TEM) and scanning transmission electron microscopy (STEM) using a Titan operating at an accelerating voltage of 300 kV. Energy-dispersive X-ray spectroscopy (EDX) were performed at 300 kV with an Oxford instruments X-max silicon Drift Detector. The data was analysed with AZtechEnergy EDX software, allowing for point scans, line scans and mapping scans to be carried out and interpreted.

#### Powder X-ray diffraction (XRD)

Powder X-ray diffraction (XRD) patterns were acquired on a Bruker D2 phaser in a 2θ range of 10°–70° using Cu Kα radiation (λ = 1.548 nm). The samples for XRD analysis were prepared by drying the sample solutions under vacuum for 2 h to get dry powder.

#### Atomic force microscopy (AFM)

The sample for AFM measurements was prepared by dispersing the QDs in toluene and dropping the sample solution onto the silicon wafers (1 cm*1 cm), which was followed by the evaporation of the solvent. The silicon wafers were washed with ethanol under sonication for 5 min beforehand. The measurement was done using AFM Asylum MFP-3D under soft tapping mode. Tips from NANOSENSORS (Type: PPP-NCST-50, S/N: 89310F7L1333) was used to do this measurement.

#### Optical spectroscopy

Samples for optical characterizations were prepared by dispersing them into toluene in 1 cm path length quartz cuvettes. UV–Vis absorption spectra were recorded using a Varian Cary 60 UV–visible spectrophotometer. PL characterization was carried out using a Horiba Jobin Yvon Fluorolog-3 using a Hamamastu InP/InGaAs photomultiplier (R5509-7–3). Excitation wavelength that used for the PL measurements was performed at least 20 nm below the emission range. Photoluminescence quantum yields (PLQYs) were calculated by the comparison of the PL intensity between samples and standard dyes, which have well-known PLQYs. HITCI (PLQY = 30% in ethanol) was used for the determination of the PLQY of the Cu–In–S QDs. Rhodamine 6G (PLQY = 91% in ethanol) was employed as the reference to calculate the PLQY of Cu–In–(Zn)–S/ZnS “ice-cream” like nanoheterostructure. In order to minimize the self-absorption of fluorescence, the absorbance of the samples was measured first to be 0.5 at peak excitation value, followed by 1:10 dilution, then the PL spectra of the samples were recorded. PL decay curves were performed using a time correlated single photon counting (TCSPC) spectrometer (Horiba Jobin Yvon) and the semiconductor diode laser (372 nm “Nano LED − 370”—HORIBA jobin Yvon) with pulse duration shorter than 200 ps for excitation.

## Results and discussion

### Synthesis of zinc blende Cu–In–S QDs

**Figure 1:**
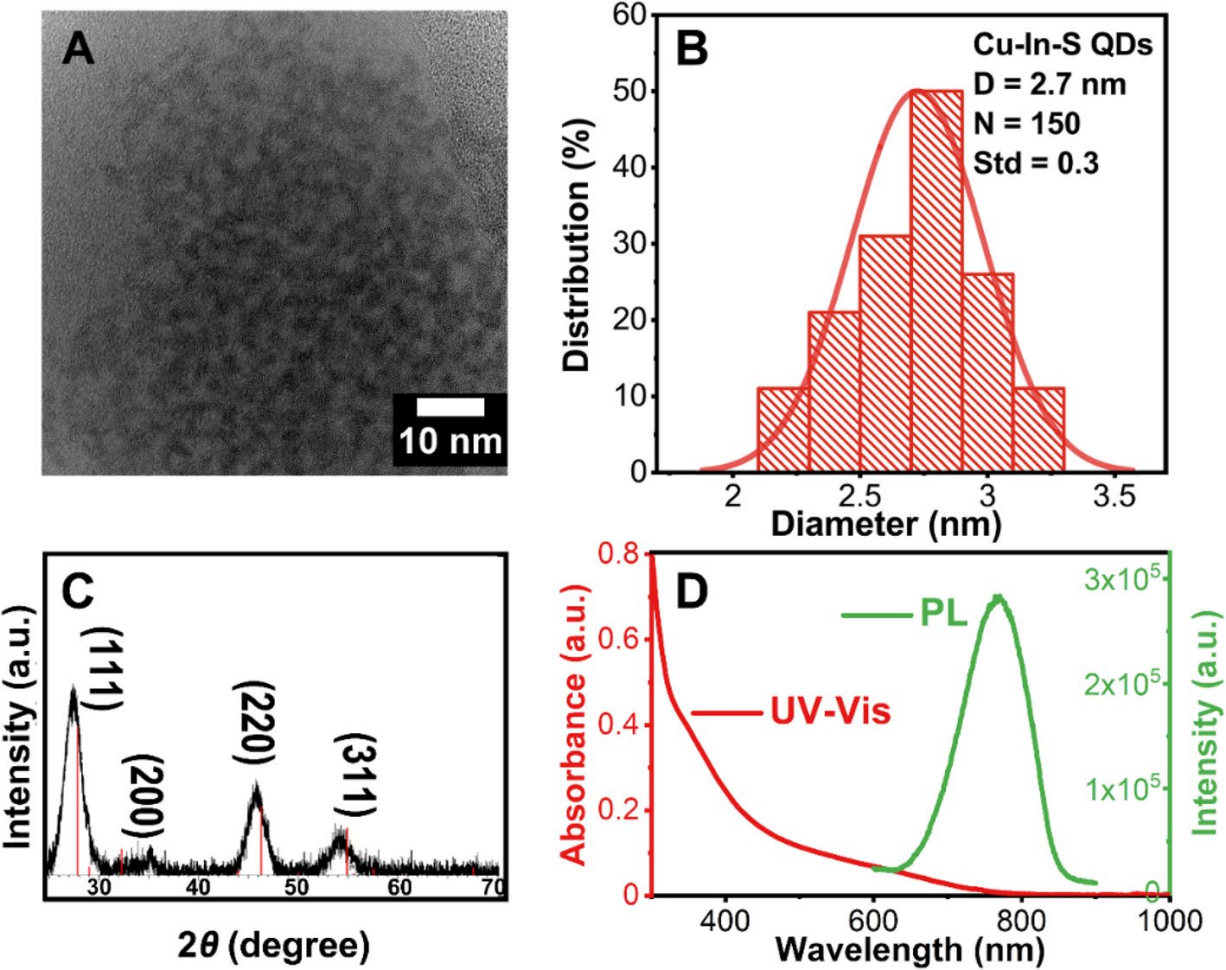
(**A**) TEM image, (**B**) Diameter distribution histogram, (**C**) XRD diffraction pattern (The red lines indicate the reference PDF 65-2732 CuInS_2_) and (**D**) UV–Vis spectrum (red line) and PL spectrum (green line) of the obtained Cu–In–S QDs.

The ZB Cu–In–S QDs were prepared using the previously reported heating-up technique, heating a mixture of CuI, In(Ac)_3_, 1-DDT and ODE to 210 °C for 2 h (see Experimental part for details)^33^. Here, 1-DDT was used as both the sulfur source and the stabilizing ligand in this reaction while ODE was used as the solvent. Figure 1A shows the TEM image of the as prepared Cu–In–S QDs, which display a spherical shape with an average diameter of 2.7 ± 0.3 nm and a narrow size distribution (Fig. 1B). The crystal structure of the obtained Cu–In–Zn–S QDs was determined using powder X-ray diffraction technique. The representing peaks of a ZB crystal structure have been identified, as shown in Fig. 1C. As shown in Fig. 1D, the UV–Vis absorption spectrum is essentially featureless, lacking the presence of any well-defined exciton absorption peak, which is a traditional trait of Cu-based multicomponent quantum dots^54–56^. It is believed that this intrinsic trait can be attributed to the existence of Cu^1+^-related defects, especially for the Cu-based QDs that are prepared with a cation ratio of Cu:In ≈ 1:1. The reason for this is due to besides the common excitonic transition from valence band to conduction band, an alternative optical transition of electrons in these QDs is possible, since the transition from Cu^1+^ to the conduction band is allowed upon excitation, which results in the extended tails reaching lower energy regions in the UV–Vis absorption spectra^57,58^. In addition, the obtained sample is fluorescent in the visible/NIR region with an emission peak centred at 770 nm and a Fwhm (full width at half maximum) of 150 nm. The PLQY of the sample was determined to be 2.7% using HITCI as the standard dye.

### Cu–In–(Zn)–S/ZnS nanocrystals after 30 min of reaction time

After Cu–In–S QDs were prepared, a ZnS shell was deposited onto the Cu–In–S seeds to improve the optical properties and stability of the sample, which was done by employing a seeded growth method (see “Experimental section” section for details). The ZnS shell precursor solution consisted of Zn(St)_2_ in a solution of OA, DDT and ODE, and was injected into the reaction vessel loaded with the Cu–In–S seeds with total injection time of 120 min. In order to monitor the evolution of the nanostructures over the reaction time and investigate the growth mechanism of their formation, we have taken aliquots from the reaction vessel for further studies after 30, 60 and 120 min.

**Figure 2:**
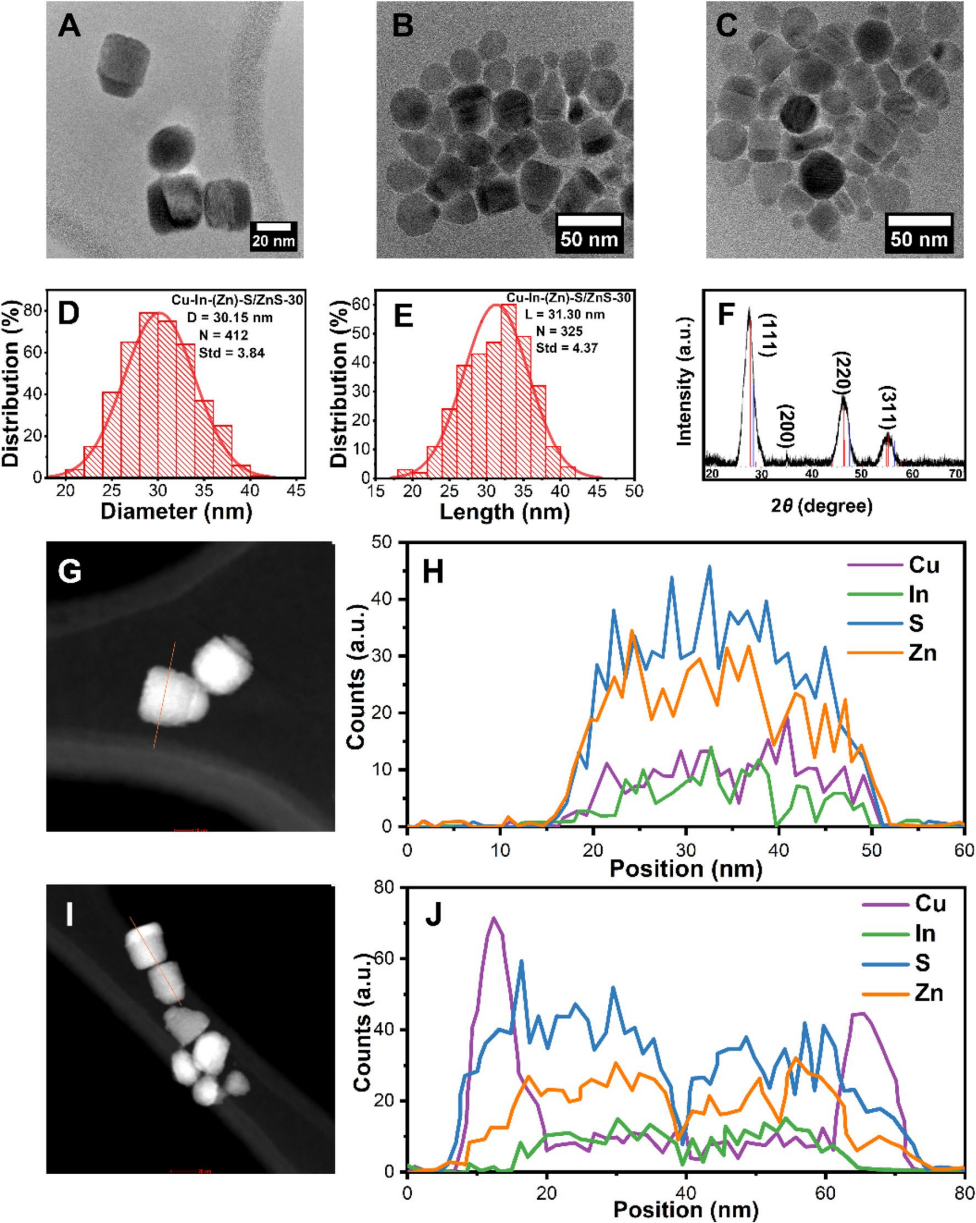
(**A**–**C**) TEM images, (**D**) Diameter histogram, (**E**) Length histogram and (**F**) XRD diffraction pattern (The red and blue lines indicate the reference PDF 47-1372 CuInS_2_ and PDF 65-1691 ZnS, respectively) of the sample Cu–In–(Zn)–S/ZnS-30. (**G**,**H**) STEM image and corresponding line scanning spectra following the direction of the orange line in (**G**). (**I**, **J**) STEM image and the corresponding line scanning spectra of the orange line in (**I**).

The sample aliquot taken out at 30 min was denoted as Cu–In–(Zn)–S/ZnS-30. As shown in Fig. 2A–C, after 30 min the majority of the particles display short cylindrical morphology with the diameter increasing to ≈ 30.2 ± 3.8 nm (Fig. 2D) from ≈ 2.7 ± 0.3 nm for the Cu–In–S seeds (Fig. 1B), and the length being calculated to be 30.3 ± 4.4 nm (Fig. 2E). The clear lattice fringes indicate the high quality of the crystal structure. A small number of cone-like structures have been observed at this time point as shown in Fig. 2B,C. The crystal structure of Cu–In–(Zn)–S/ZnS-30 is also determined to be the ZB phase (Fig. 2F) by XRD analysis. The morphology of the sample was verified further with STEM images and is shown in Fig. 2G,I (more STEM images of Cu–In–(Zn)–S/ZnS-30 can be found in Fig. S1 in Supporting information).

EDX line scanning has been performed on Cu–In–(Zn)–S/ZnS-30 to determine the elemental distribution along two different directions, parallel to the top and bottom surfaces of the cylindrical structure and perpendicular to the top and bottom surfaces, respectively. Interestingly, different elemental distribution trends can be observed along the two different scanning directions. The line scanning spectra shown in Fig. 2H was carried out along the direction parallel to the top and bottom surfaces of the cylinder (marked with orange line in Fig. 2G). It was found that elements Cu and In are distributed evenly along the scanning direction instead of being confined in a small spot in this structure, considering the small size of the Cu–In–S core compared to the much larger size of Cu–In–(Zn)–S/ZnS-30. It is expected that elements Zn and S can be found along the whole scanning range since the two elements are from the ZnS shell, while the even distribution of elements Cu and In instead of being confined in one small spot is an excellent indication that in fact an alloyed Cu–In–(Zn)–S/ZnS structure was formed at this stage, which is a common phenomenon during ZnS shell coating^59–64^. The interdiffusion between the core and the shell was also reported by Min Fu et al. using inductively coupled plasma-optical emission spectroscopy and X-ray photoelectron spectroscopy^65^. Here, the high reaction temperature (230 °C) and the similar size of elements Zn, Cu and In facilitated Zn diffusion into the Cu–In–S core, which induced the formation of the alloyed Cu–In–(Zn)–S/ZnS structure. The intensities of elements Zn and S are much higher than that of elements Cu and In (Fig. 2H), which is due to the large volume ratio between the much larger Cu–In–(Zn)–S/ZnS-30 (diameter ≈ 30 nm) core/shell structure and the original small Cu–In–S seeds (diameter ≈ 3 nm). In contrast, it is a completely different case when the line scanning was carried out along the direction perpendicular to the top and bottom surfaces of the cylinder (indicated by the orange line in Fig. 2I). As shown in Fig. 2J, the intensities of Zn and S are higher due to the much greater size of nanostructures upon the incorporation of the elements. Interestingly, the intensity of Cu is much higher at one side (1/4 of the total length of the cylinder) than the opposite side (the remaining 3/4) of the cylinder and indicates that more Cu is located in this 1/4 part of the cylindrical structure than the amount of Cu in the remaining 3/4 part (line scanning has been carried out upon multiple particles as shown in Fig. S2A–D in Supporting information). In addition, two adjacent Cu–In–(Zn)–S/ZnS-30 nanoparticles were scanned at the same time, resulting in a relatively symmetrical distribution of Cu intensities. Additionally, the heavier distribution of Cu at one side of the cylinder accounted for the intensity contrast that is shown in the TEM images (Fig. 2A–C, darker side of the cylinder) and STEM images (Fig. 2G,I, brighter side of the cylinder). The occurrence of Zn and S outside the alloyed Cu–In–(Zn)–S part observed in Fig. 2H,J indicates that an alloyed Cu–In–(Zn)–S structure has been obtained with a thin ZnS shell forming on the outside after 30 min of the reaction.

### Cu–In–(Zn)–S/ZnS nanocrystals after 60 min of reaction time

**Figure 3:**
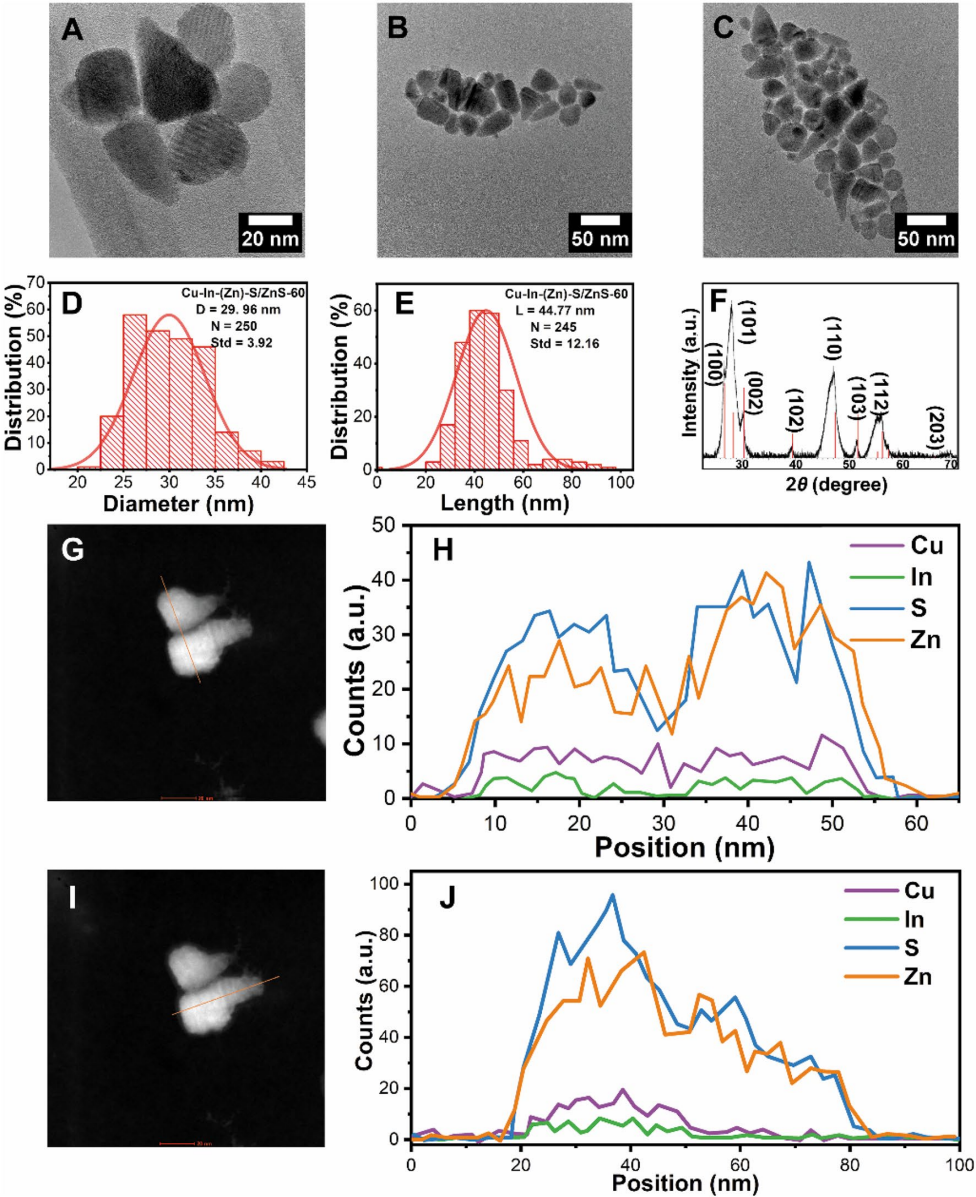
(**A**–**C**) TEM images, (**D**) Diameter histogram, (**E**) Length histogram and (**F**) XRD diffraction pattern (The red lines indicate the reference PDF 75-1547 ZnS) of the sample Cu–In–(Zn)–S/ZnS-60. (**G**,**H**) STEM image and corresponding line scanning spectra following the direction of the orange line in (**G**). (**I**, **J**) STEM image and the corresponding line scanning spectra of the orange line in (**I**).

An aliquot after 60 min was also taken out from the reaction vessel for characterization, which is denoted as Cu–In–(Zn)–S/ZnS-60. Figure 3A–C shows the TEM images demonstrating that the nanoparticles with the cone-like morphology clearly dominated in occurrence as the reaction proceeds. The diameter of the structure maintained the same relative value (≈30.0 ± 3.9 nm, Fig. 3D), while the length of Cu–In–(Zn)–S/ZnS-60 increased to 44.8 ± 12.2 nm (Fig. 3E) from 31.3 ± 4.4 nm of Cu–In–(Zn)–S/ZnS-30 (Fig. 2E). The crystal structure was determined to be WZ of Cu–In–(Zn)–S/ZnS-60 (Fig. 3F). In addition, the intensity contrast across particles observed for Cu–In–(Zn)–S/ZnS-30 are not clearly observable for Cu–In–(Zn)–S/ZnS-60. EDX line scanning has been performed on Cu–In–(Zn)–S/ZnS-60 to verify the observation from TEM images. As shown in Fig. 3G, the intensity contrast can hardly be observed. The line scanning spectra (Fig. 3H) of the two adjacent particles along the direction indicated with the orange line in Fig. 3G confirmed the even distribution of elements Cu and In in both particles along this direction. The line scanning spectra displayed in Fig. 3J along the direction indicated with the orange line in Fig. 3I demonstrates that elements Cu and In are confined in the “head” of the “ice-cream cone” like structure. These two elements can also be observed to distribute throughout this “head” part evenly, in contrast to the uneven distribution of Cu and In after 30 min. The “cone” part of the “ice-cream cone” like structure consists of elements Zn and S, while elements Cu and In can scarcely be detected.

### Cu–In–(Zn)–S/ZnS “ice-cream cone” like nanoheterostructures

**Figure 4:**
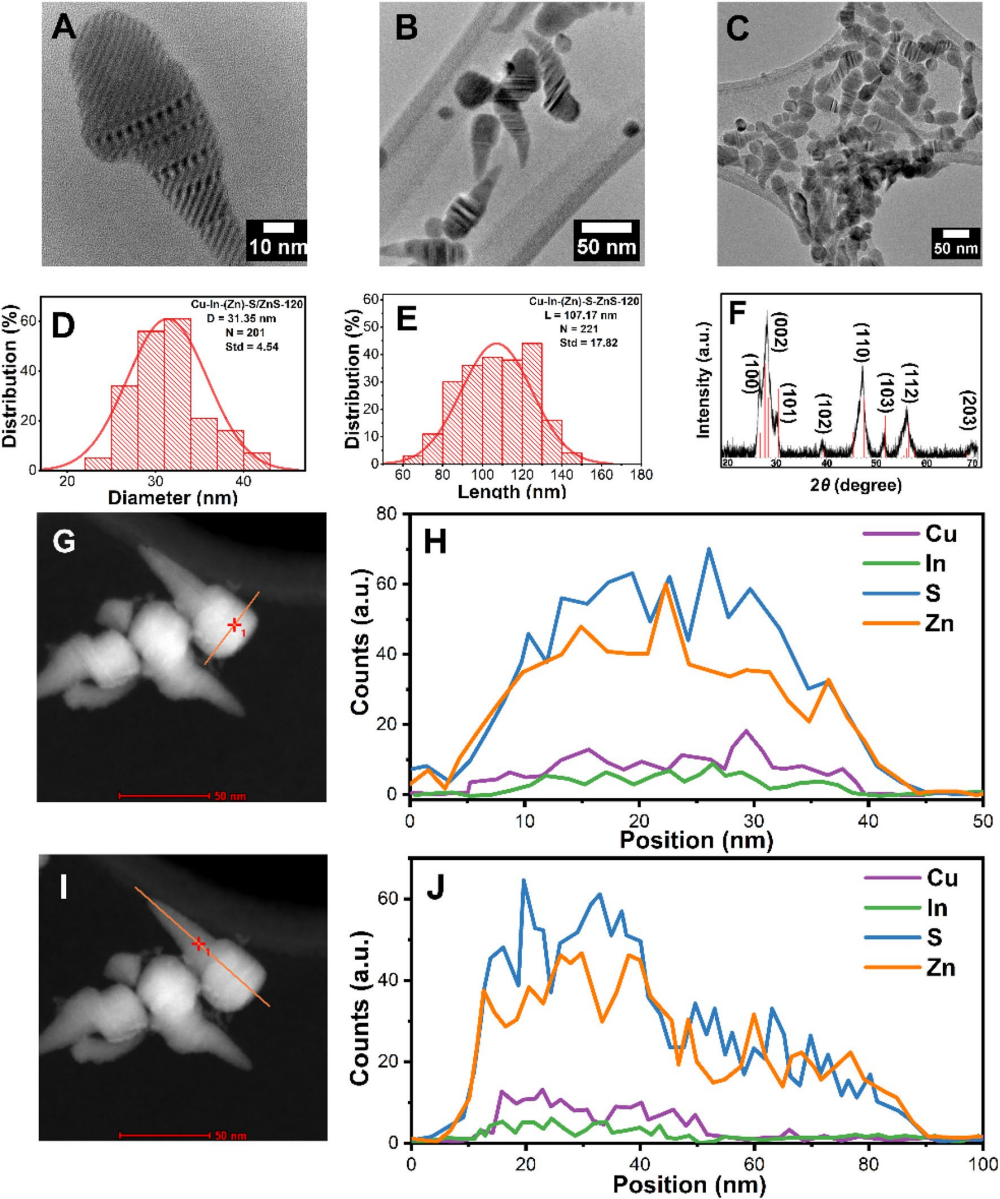
(**A**–**C**) TEM images, (**D**) Diameter histogram, (**E**) Length histogram and (**F**) XRD diffraction pattern (The red lines indicate the reference PDF 89-7334 ZnS.) of the sample Cu–In–(Zn)–S/ZnS-120. (**G**,**H**) STEM image and corresponding line scanning spectra following the direction of the orange line in (**G**). (**I**,**J**) STEM image and the corresponding line scanning spectra of the orange line in (**I**).

Injection of the mixture of Zn(St)_2_ and 1-DDT as the ZnS shell precursor solution was completed after 120 min, and the sample Cu–In–(Zn)–S/ZnS-120 has been characterized by electron microscopy and spectroscopy techniques. The nanostructures with a cone-like morphology observed in Cu–In–(Zn)–S/ZnS-60 have been transformed into the final “ice-cream cone” like nanostructures according to the TEM images shown in Fig. 4A–C (more TEM images can be found in Fig. S3A–C in Supporting information). The diameter remained relatively similar to Cu–In–(Zn)–S/ZnS-60 (≈ 31.4 ± 4.5 nm, Fig. 4D), while the length of the “ice-cream cone” like nanostructure significantly increased and was determined to be ≈ 107.2 ± 17.8 nm (Fig. 4E). In addition, using XRD it was revealed that the “ice-cream cone” like nanostructures demonstrate the WZ crystal structure (Fig. 4F).

The morphology and size of this structure was confirmed by STEM imaging (Fig. 4G,I, more STEM images can be found in Fig. S4 in Supporting information), while also EDX line scanning was conducted to determine the elemental distribution in this unique nanostructure (Fig. 4H,J). Firstly, all the expected elements (Cu, In, Zn and S) can be easily detected in the structure, with the intensities of Cu and In significantly lower than that of the other elements detected, which is due to the small volume of the original Cu–In–S core relative to the shell. In addition, the even distribution of Cu and In are confined to the “head” of the “ice-cream cone” like nanostructure. Therefore, this clearly indicates the position of the alloyed Cu–In–(Zn)–S core. Observing the trends of the shell material, Zn and S, they presented from the “head” part to the “cone” part of the structure. In total, the final “ice-cream cone” like nanoheterostructure consists of the Cu–In–(Zn)–S/ZnS “head” part and the ZnS “cone” part.

The final “ice-cream cone” like nanoheterostructure has also been studied using AFM. The phase and topography images confirmed the morphology of the “ice-cream” like geometry laying on the silicon wafer (Fig. S5A–C in Supporting information). The average length ≈ 110 nm measured from the elongated “ice-cream cone” like structure in AFM image (Fig. S5D in Supporting information) is consistent with the lengths obtained from TEM images. The sample demonstrated high colloidal stability and the PL from the sample has been kept even after long term storage (the pictures that taken under daylight and UV lamp on the sample after being stored in the fridge for 21 months can be found in Fig. S6).

### Spectroscopic characterization

**Figure 5:**
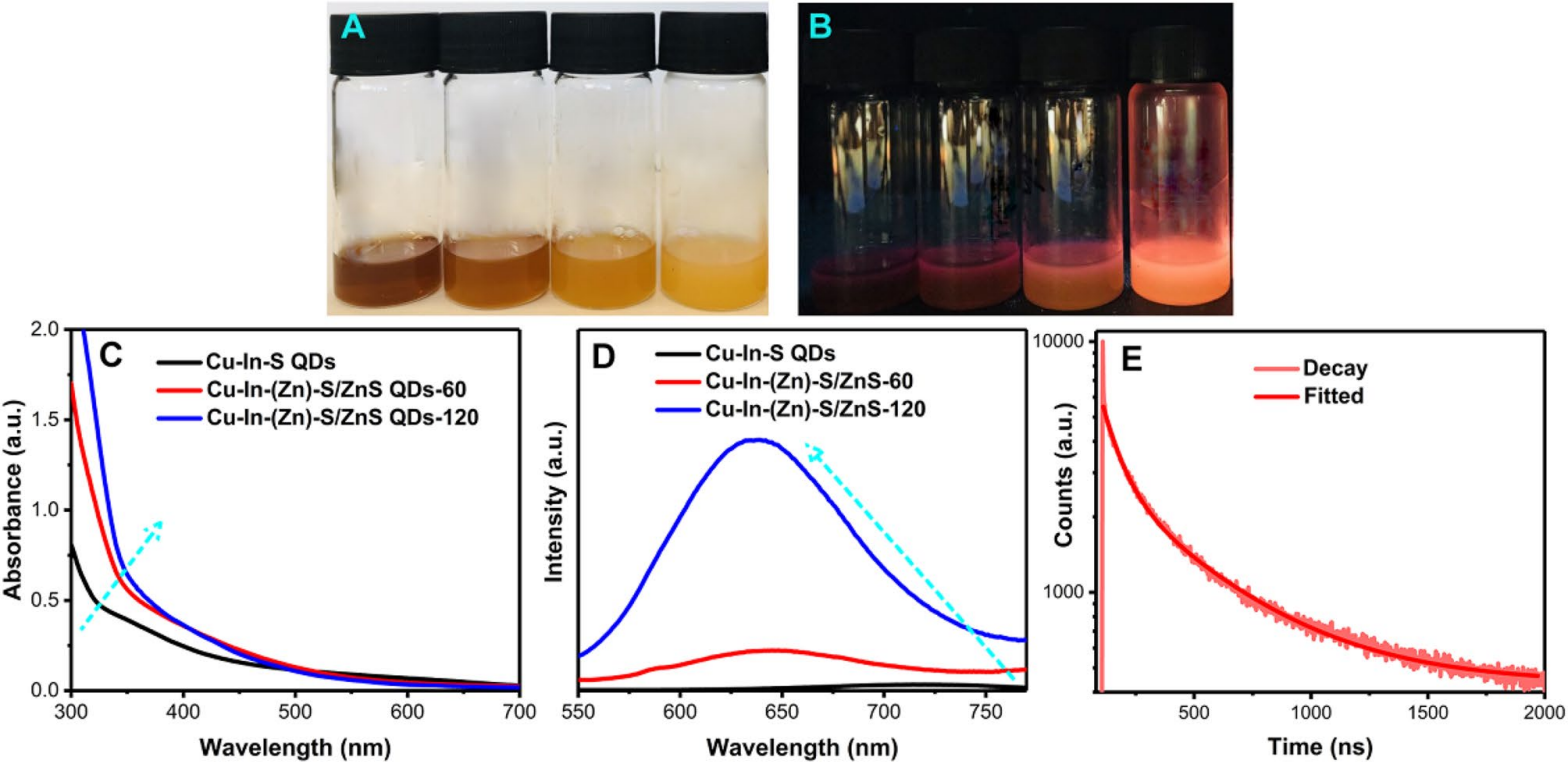
(**A**,**B**) Photographs of aliquots taken from the reaction vessel at different time intervals (from left to right, 10 min, 30 min, 60 min and 120 min) under daylight and UV lamp. (**C**,**D**) UV–Vis spectra and PL spectra of Cu–In–S QDs, Cu–In–(Zn)–S/ZnS-60 and Cu–In–(Zn)–S/ZnS-120, respectively. (**E**) PL decay curve of Cu–In–(Zn)–S/ZnS-120.

A series of spectroscopic characterizations have been carried out on 4 aliquots (after 10, 30, 60 and 120 min of the reaction process). As shown in Fig. 5A, the colour of the aliquots changed from brown (10 min) to bright orange (120 min) under daylight. Under a UV lamp, the sample shows increasing luminescence with the reaction time (Fig. 5B). Figure 5C shows the UV–Vis spectra of the Cu–In–S QDs, the sample Cu–In–(Zn)–S/Zn–S-60 and the sample Cu–In–(Zn)–S/ZnS-120. Due to the traditional trait of multicomponent Cu-based quantum dots, these UV–Vis spectra are featureless without any observable well-defined exciton absorption peaks for all three samples^54–56^. The enhanced absorbance and the blue shift of the onset of the absorption before 400 nm can be observed as the reaction proceeds, which is due to the increased absorption cross-section upon coating with ZnS, a wide band gap semiconductor. Therefore, the Cu–In–(Zn)–S/ZnS “ice cream cone” heterostructures represent an effective down-converting luminescent NC.

The corresponding photoluminescence (PL) spectra of the three samples are shown in Fig. 5D. The Cu–In–S QDs are emissive in the NIR region (770 nm) with relatively low PLQY (2.7%). After 60 min of ZnS shell precursor solution injection, a substantial blue shift (125 nm) can be observed, shifting the emission peak from 770 nm for the Cu–In–S core to 645 nm for Cu–In–(Zn)–S/ZnS-60. When the reaction reached completion, the emission peak of Cu–In–(Zn)–S/ZnS-120 showed a further minor blue shift (7 nm) from 645 to 638 nm. Such an observed blue shift of PL peaks have commonly been reported during the synthesis of Cu-based quantum dots^42,66^ particularly for Cu-based ternary or quaternary core/shell nanostructures involving Zn^60,61,66–69^. It was found that Zn(St)_2_ was the most effective precursor for achieving a blue shift among a range of Zn precursors for the preparation of CuInS_2_/ZnS core/shell quantum dots, including zinc chloride, zinc acetylacetonate and zinc acetate reported by Jaehyun park and Sang-Wook Kim^61^. The mechanisms underlying this PL blue-shift phenomena during the synthesis are not clearly established, with several pathways having been proposed such as: (1) Filling up of intra-bandgap defect states^65,70^, (2) Core size reduction by cation exchange^59–61,71^ and (3) Band gap widening by the entrance of Zn ions^72^. The first mechanism is mainly about passivation by means of ZnS shelling, which facilitates the reduction of the fraction of donor–acceptor recombination pathways with lower energy levels; thus the band-edge or band to donor/acceptor emission with higher energy levels becomes the dominant emissive deexcitation pathway, leading to the observed blue shift. Core size reduction by cation exchange^59–61,71^ is proposed to occur due to the similar sizes of the metal cations present of Cu, In and Zn. Since the reaction takes place at high temperatures, Zn^2+^ can diffuse into the Cu-based ternary or quaternary core lattice structure and can replace the Cu^1+^ and In^3+^ ions, therefore reducing the size of the core and resulting in core-size dependent blue shift. Finally, band gap widening by the entrance of Zn ions into the lattice^72^ can take place via diffusion of them into the pre-existed Cu-based seeds. This induces a widening of the band gap, which is quite similar to the doping of Zn into CIS thin films^73^.

In this work, it was verified that alloyed Cu–In–(Zn)–S core formed through the entrance of Zn into the original Cu–In–S seeds (Fig. 2D,F) as the reaction proceeds. In addition to the formed alloyed structure, ZnS shell has been deposited onto the alloyed core part. Thus, it can be proposed that the combined effects of above-mentioned factors (1) and (3) induced this dramatic PL blue shifting as the reaction proceeds at high reaction temperature (230 °C) for significant reaction time (120 min). As the ZnS shelling proceeds, the intensity of the emission greatly increases, giving a significant increase in PLQY of the final Cu–In–(Zn)–S/ZnS “ice-cream cone” like structure up to 27.7 ± 2.8%. This is the highest PLQY reported to date for a Cu–In–(Zn)–S/ZnS anisotropic nanoheterostructure, with the previous record being 20%^74^. Based on the obtained multiple TEM images of the sample (Fig. S7), the percentage of the anisotropic elongated nanostructure among all the other morphologies is 70%. It can be concluded that the PL from the elongated anisotropic structures predominates the total emission of the whole batch of the sample. The gradually improved PL properties can be interpreted to be due to the surface passivation effect of the ZnS shell upon the surface defects which can act as a nonradiative pathway^39–41^. PL decay curve of the final Cu–In–(Zn)–S/ZnS “ice-cream cone” structure, which was biexponentially fitted is shown in Fig. 5E and the average PL lifetime was found to be 366 ns (see the two lifetime components in Table S1). Noteworthy is the distinct advantage of such a long PL lifetime which could be especially suit to bioimaging^75^.

**Figure 6:**
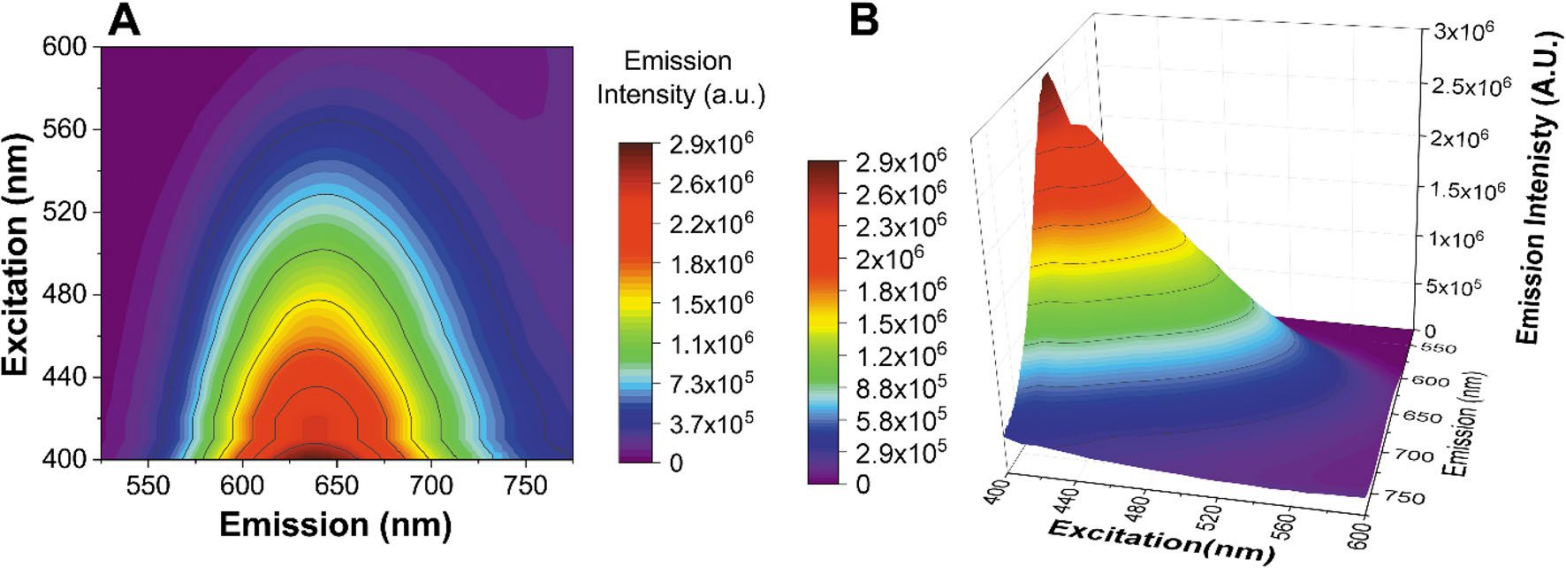
EEM (excitation emission matrix) of Cu-In-(Zn)-S/ZnS-120.

The EEM (Excitation Emission Matrix) of Cu-In-(Zn)-S/ZnS-120 is shown in a contour map and as a 3-dimensional plot in Fig. 6A,B respectively. These spectra were adjusted with a correction factor so as to take account of the inner filter effect (calculated using Equations S1 and S2 in supporting information), in addition lamp excitation peaks have been removed from the spectra. The resulting spectra of Fig. 6A,B show a distinctive behavior of the material, showing a strong maximum emission intensity in the range of 630–640 nm upon excitation of approximately 400–410 nm and a general decrease in emission intensity with lower energy excitation. In addition, no peak maximum red shifting is found to occur upon excitation variation, which again supports the lack of a broad size distribution of the “ice-cream cone” sample.

### Mechanism of morphological development of the “ice-cream cone” nanoheterostructures

It is widely reported that the energy barrier for heterogeneous nucleation is much lower than homonucleation if the lattice mismatch between the seeds and the nuclei is not substantial^8,76–78^. Therefore, under these conditions, homonucleation of the shell material precursors will be unfavourable and instead heteronucleation will dominate, enabling the controlled growth of heteronanostructures in this case. The seeded growth technique takes full advantage of this approach and has been widely used to synthesize both Cd-based and Cu-based ternary or quaternary core/shell heterostructures with various morphologies (spherical, dot-in-rod, tetrapod or even rod-in-rod structures)^14,15,74,79^. Therefore, this technique has been employed here to prepare the unique “ice-cream cone” nanoheterostructure.

**Figure 7:**
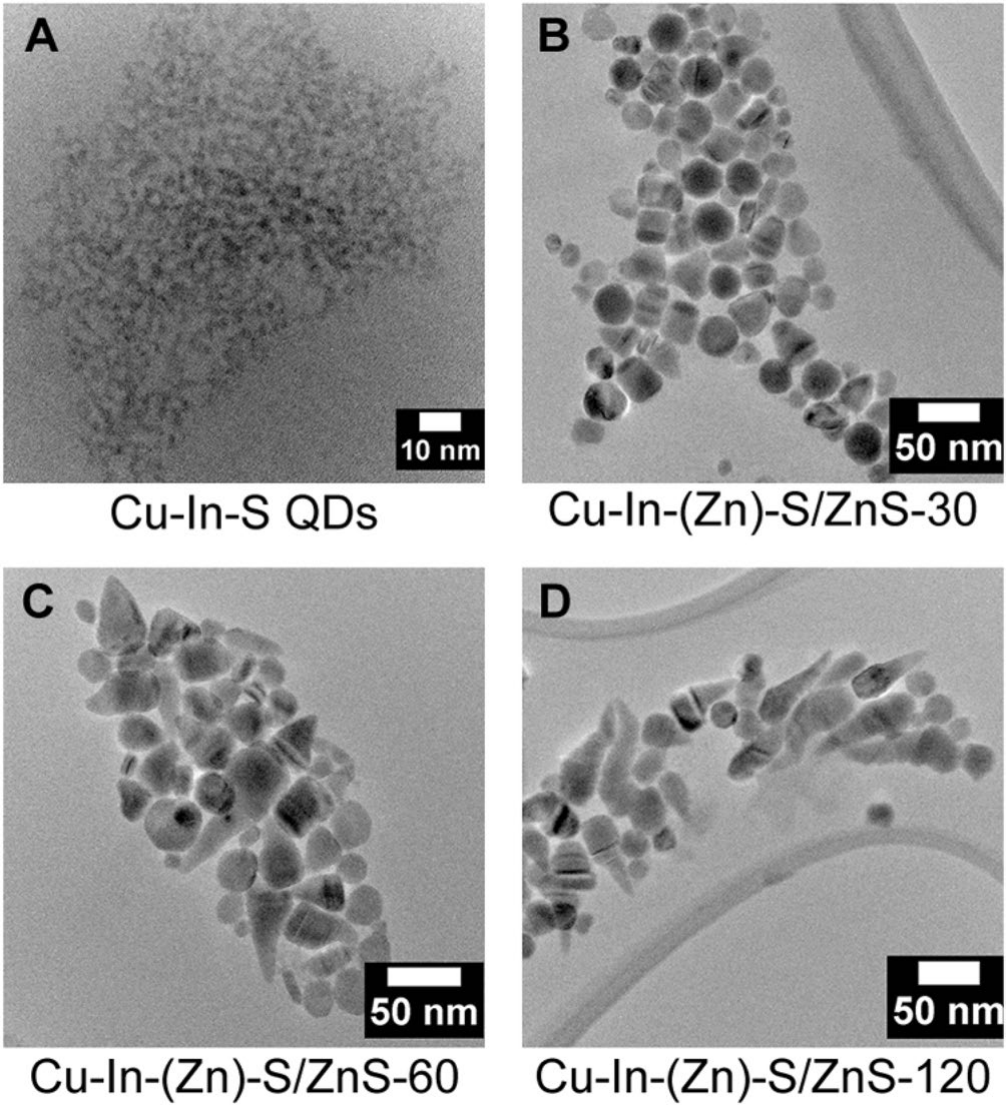
(**A**–**D**) TEM images showing the morphology development of the Cu–In–(Zn)–S/ZnS “ice-cream” like nanostructures.

The preformed ZB Cu–In–S QDs (≈ 3 nm) served as the seeds for heterogeneous nucleation and heteroepitaxial growth (Fig. 7A) of the “ice-cream cone” nanoheterostructures. The size of the sample increased progressively as the reaction proceeds, with a spherical morphology present after 5 min ZnS shell growth, measuring 20.8 ± 2.1 nm in diameter progressing to 29.4 ± 2.9 nm dimeter after 15 min growth (see Fig. S8 in Supporting information for details of the samples that taken out from the reaction vessel at 5 and 15 min).

A blank experiment was conducted by mixing the precursors of the core and the shell together in a reaction vessel and heating the mixture up to 230 °C for 2 h (see details in supporting information). As shown in Fig. S9, the obtained QDs are in spherical shape (≈ 2.6 nm) without the presence of the Cu–In–S seeds in the reaction vessel.

We intended to investigate the effect of the size of the Cu–In–S QDs on the morphology of the final core/shell heterostructure. A series of reactions have been carried out trying to prepare Cu–In–S seeds with quite large size differences by extending the reaction time from 2 to 4 h at 210 °C and increasing the reaction temperature from 210 to 230 °C. As show in Table S2 and Fig. S10, the sizes of the obtained 3 batch of samples are in a quite limited tuning range from 2.7 to 3.3 nm.

After 30 min of ZnS shell growth, the NC transformed from a spherical morphology to a cylindrical core/shell structure and size increased significantly as shown in Fig. 7B. While the crystal structure remained the same (ZB crystal structure) for the sample at 30 min. Interestingly, the EDX line scanning spectra (Fig. 2J) showed that the elements Cu and In were distributed unevenly along the direction perpendicular to the top and bottom surfaces with more Cu located at the first 1/4 and In distribution at the remaining 3/4 part of the cylindrical structure. In fact the seeded growth method is known to prepare core/shell heterostructures, in which the cation precursors of the shell can enter the pre-existing cores through interdiffusion/substitution processes, with the rate of this highly dependent on reaction conditions^31,72,80^. In this case, the higher reaction temperature (230 °C) and similar sized metal cations (Cu, In and Zn, cation radius = 60–62 pm in a four coordinate system)^61,66^ promote the interdiffusion process giving rise to the formation of the alloyed Cu–In–(Zn)–S/ZnS structure as opposed to the Cu–In–S /ZnS core/shell structure.

After 60 min of shell growth, the sample became cone-like (Fig. 7C) and the crystal structure transformed from ZB to WZ. While after 120 min, the sample consisted nearly entirely of the “ice-cream cone” like structures (Fig. 7D). The EDX line scanning spectra (Fig. 4J) revealed that elements Cu and In were detected at the top part of the “ice-cream cone” nanoheterostructure, which indicates that the Cu–In–(Zn)–S core is located at the “head” of the “ice-cream cone”. The extended growth of a ZnS shell results in the formation of this unique nanostructure.

The formation of the elongated “ice-cream cone” like structure can thus be interpreted by considering the evolution of the crystal structure from ZB to WZ as the reaction proceeds. The ZB crystal structure was maintained from the Cu–In–S seeds to the sample Cu–In–(Zn)–S/ZnS-30, which was followed by the transformation of the crystal structure from ZB to WZ. Then the “ice-cream cone” like structure started to occur. The wurtzite QDs have two polar directions [0001] and [000-1]. The reactivity towards the [000-1] direction is higher than that towards the [0001] direction due to cations towards [000-1] direction expose more (three) dangling bonds than the cations towards [0001] direction (only one). The shell materials prone to attach on these polar facets compared with other non-polar facets^9^. The growth of the ZnS shell was proceeded via the addition of the [ZnS] monomer onto the facet (it was also claimed for the Cd-based anisotropic nanorod structure)^8,50,74,81–83^, maintaining the polarity of the growing facet and resulted in the cylindrical nanoheterostructures. Since the [000-1] expose more dangling bonds, the growth rate along this direction is higher than that towards the opposite direction. It has been found that the wurtzite cylindrical structures tend to develop into asymmetric elongated morphology along the c-axis with a plat end and a tapering tail after they grow to a certain length^3,84^. This phenomenon has not only observed in nanorod structures but also in octapod-shaped nanostructures^1^. The reactions to prepare QDs, especially the ones involving higher reaction temperatures and inert atmosphere, are prone to proceed in a way to realize a lower energy of the entire reaction system. As the reaction proceeds, the polar facets with more dangling bonds can be replaced by either polar facets with lower dangling bonds or non-polar facets. Later on, Bertoni et al. have carried out detailed work to investigate the polarity and faceting of wurtzite CdSe/CdS nanorods^85^. The {10-1-1} surfaces, forming the tip along [000–1] direction have been confirmed by the angles via atomic column projections. The Cd atoms only expose one and a half dangling bonds on average on the oblique planes and these findings can be applied to Cu-based QDs. The cations (Cu, In and Zn) on the formed oblique planes from these higher index facets expose less dangling bonds compared to that of the (000-1) surface, which means that less ZnS shell material can be deposited on these facets and results in the observed tapering tail.

## Conclusions

A seeded growth method has been used to produce a unique Cu-based multicomponent Cu–In–(Zn)–S/ZnS “ice-cream cone” like nanoheterostructure with emission in the visible region and high PLQY. Using detailed time studies, we have demonstrated the evolution of this morphology, studying the resulting crystal structure, nanocrystal morphology and optical properties at multiple stages of the synthesis. From this we have identified several impactful results.

Focusing on optical properties, we have demonstrated that due to a combination of effects including band gap widening by the entrance of Zn ions into the core and the passivation of intragap defect states, a significant blue shift of the PL peak from the NIR region (770 nm) of the Cu–In–S core to the visible region (638 nm) of the final Cu–In–(Zn)–S/ZnS “ice-cream cone” like nanoheterostructure can be seen. In addition, across this same time we find a significant improvement in emission intensity, giving a final PLQY of up to 27.2%, the highest PLQY reported to date for a Cu–In–(Zn)–S/ZnS anisotropic nanoheterostructure.

Using STEM and TEM we have shown the evolution of the NC morphology. Beginning with Cu–In–S QDs, an initial short cylindrical NC after 30 min is produced, which becomes a cone shaped NC after 60 min, to become the distinct final ‘ice-cream cone’ NC morphology after 120 min of ZnS shell growth. Over this time, we find from the initial Cu–In–S QDs of 2.7 ± 0.3 nm, we end with a large Cu–In–(Zn)–S/ZnS “ice-cream cone” like nanoheterostructure measuring 31.4 ± 4.5 nm in diameter with a length of ≈ 107.2 ± 17.8 nm. This enormous change in size and shape is an important finding and has been achieving through careful experimental design to produce a bespoke NC structure never seen before.

The growth mechanism of this “ice-cream cone” like geometry was therefore examined in detail using EDX and XRD in combination with the electron microscopy imaging. From this we have been able to show that the evolution of crystal structure from ZB to WZ as the reaction proceeds, plays a key role and eventually results in the elongated “ice-cream cone” like anisotropic shape. This is an impactful finding and displays the power of using multiple approaches to fully understand the evolution of unique NC morphology in the Cu-ternary semiconductor NCs.

Overall, this research offers new methodology and possibilities to prepare unique fluorescent non-toxic Cu–In–Zn–S based multicomponent anisotropic heteronanostructures, while also offering a distinctive insight into the design of bespoke nanostructures. In addition, this work offers an important methodology to design and prepare new unique highly luminescent Cu-based multicomponent anisotropic nanostructures which could find potential applications in photonics, photovoltaics, optoelectronics, imaging, and sensing.

## Supplementary Information

Supplementary Information.
