# Supplementary material for: Photoluminescent, “ice-cream cone” like Cu–In–(Zn)–S/ZnS nanoheterostructures
Source: Sci Rep. 2022 Apr 6;12:5787. doi: 10.1038/s41598-022-09646-3 (PMC8987046; doi:10.1038/s41598-022-09646-3)
Supplement: Supplementary file 1 — Supplementary Information. [file 41598_2022_9646_MOESM1_ESM.docx]

Supporting Information

Photoluminescent, “Ice-Cream Cone” like Cu-In-(Zn)-S/ZnS Nanoheterostructures

Xue Bai^a^, Finn Purcell-Milton^a^, Daniel K. Kehoe^a^ and Yurii K. Gun;ko*^a^

^a^School of Chemistry and CRANN institute, Trinity College Dublin, Dublin 2, Dublin, D02, Ireland.

E-mail: [IGOUNKO@tcd.ie](mailto:IGOUNKO@tcd.ie)


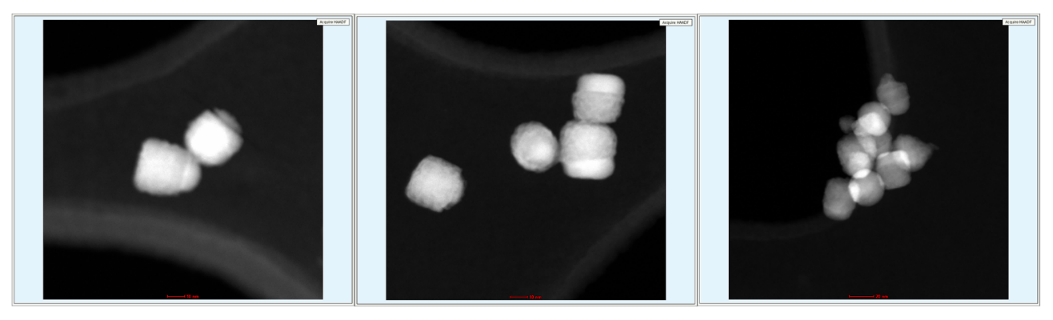


**Figure S1.** STEM images of Cu-In-(Zn)-S/ZnS-30.


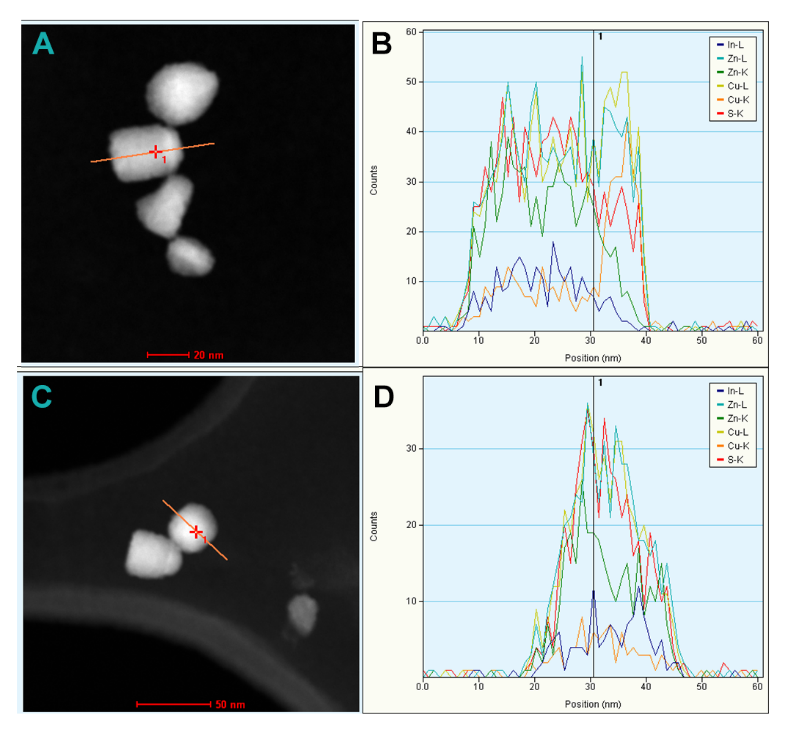


**Figure S2.** (A) STEM image of Cu-In-(Zn)-S/ZnS-30 and (B) the corresponding line scanning spectra of the particle in Figure S2A along the direction that indicated with the yellow line (perpendicular to the top and bottom surfaces of the cylindrical structure). (C) STEM image of Cu-In-(Zn)-S/ZnS-30 and (D) the corresponding line scanning spectra of the particle along the direction that indicated with the yellow line in Figure S2C (parallel to the top and bottom surfaces of the cylindrical particle).


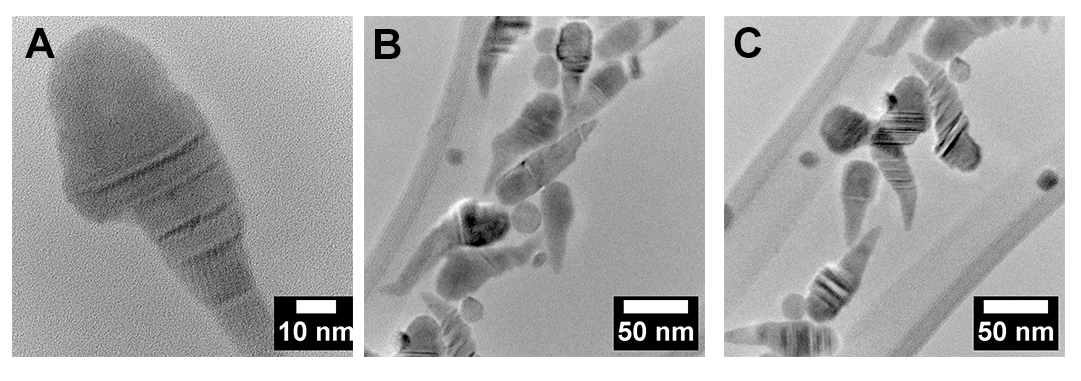


**Figure S3.** (A-C) TEM images of the final Cu-In-(Zn)-S/ZnS “ice-cream cone” like nanoheterostructure.


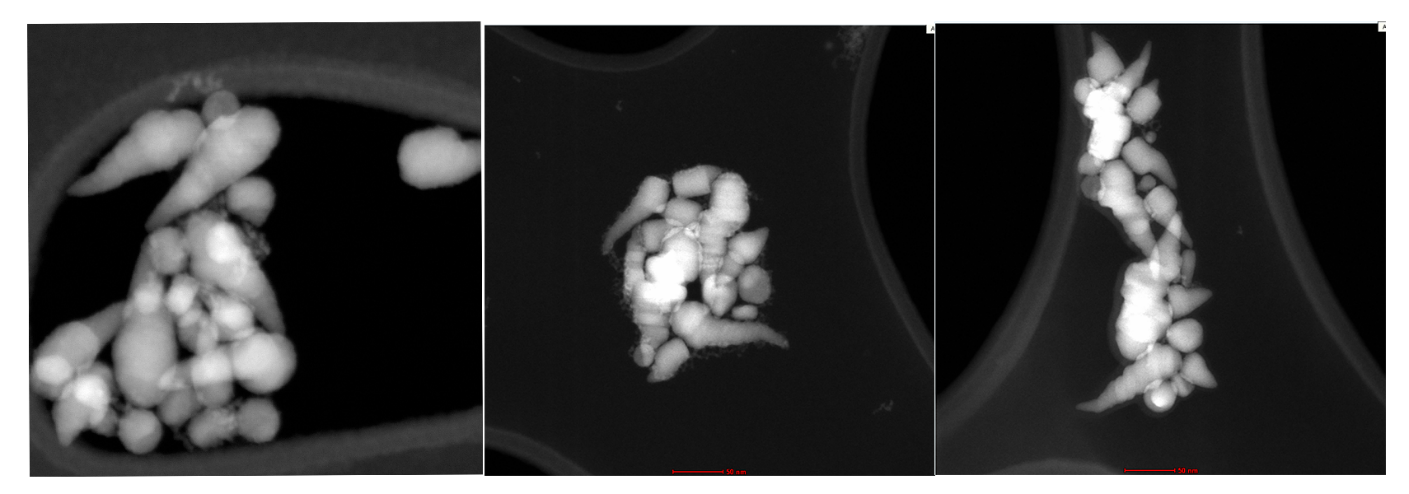


**Figure S4.** STEM images of the final Cu-In-(Zn)-S/ZnS “ice-cream cone” like nanoheterostructure.


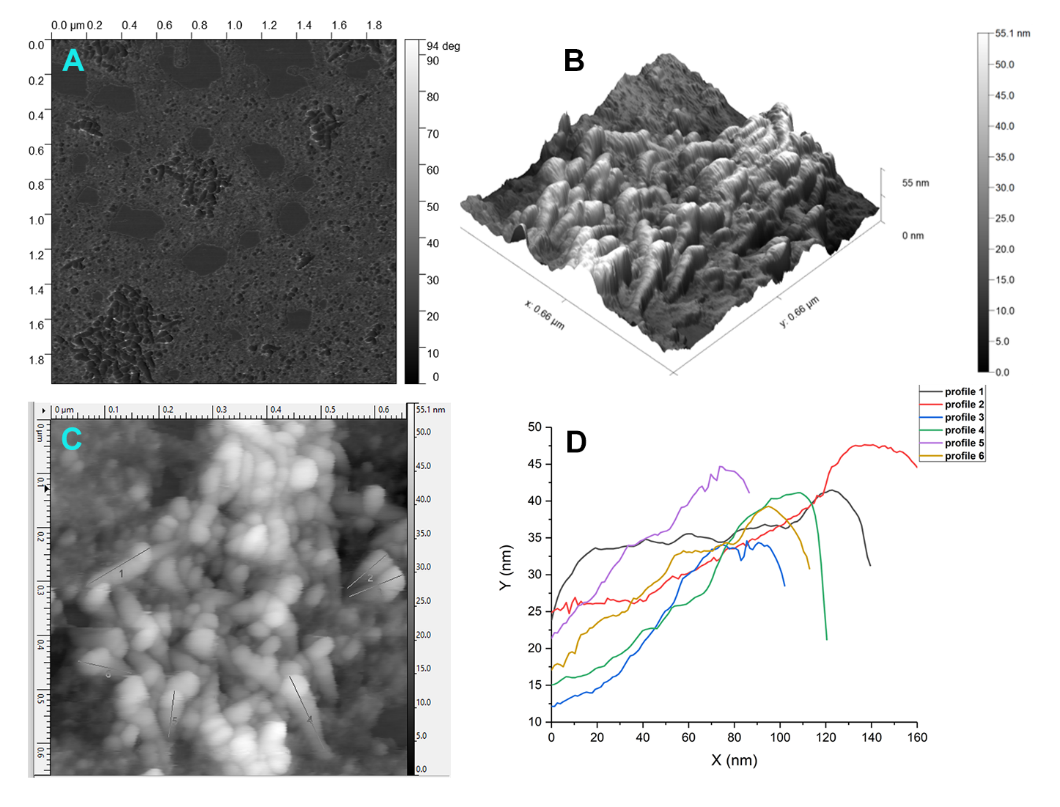


**Figure S5.** (A) Phase image and (B) the corresponding topography image, (C) enlarged Phase image of the final Cu-In-(Zn)-S/ZnS “ice-cream” like nanostructure, (D) the morphological information (length and thickness) measured from the structures shown in Figure S5C.


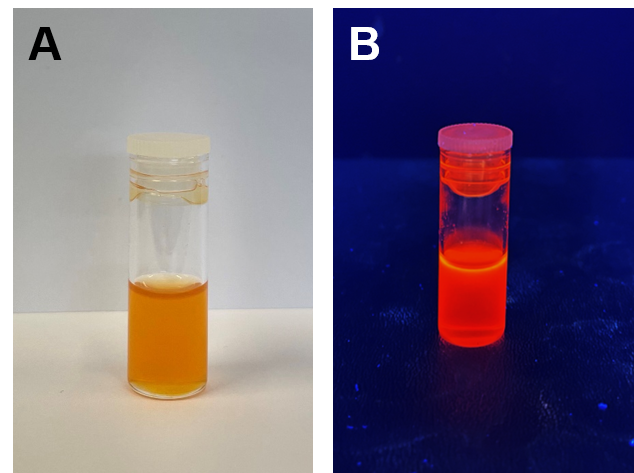


**Figure S6**. The pictures of the obtained Cu-In-(Zn)-S/ZnS “ice-cream cone” like structures under (A) day light and (B) UV lamp after being stored in the fridge for 21 months.


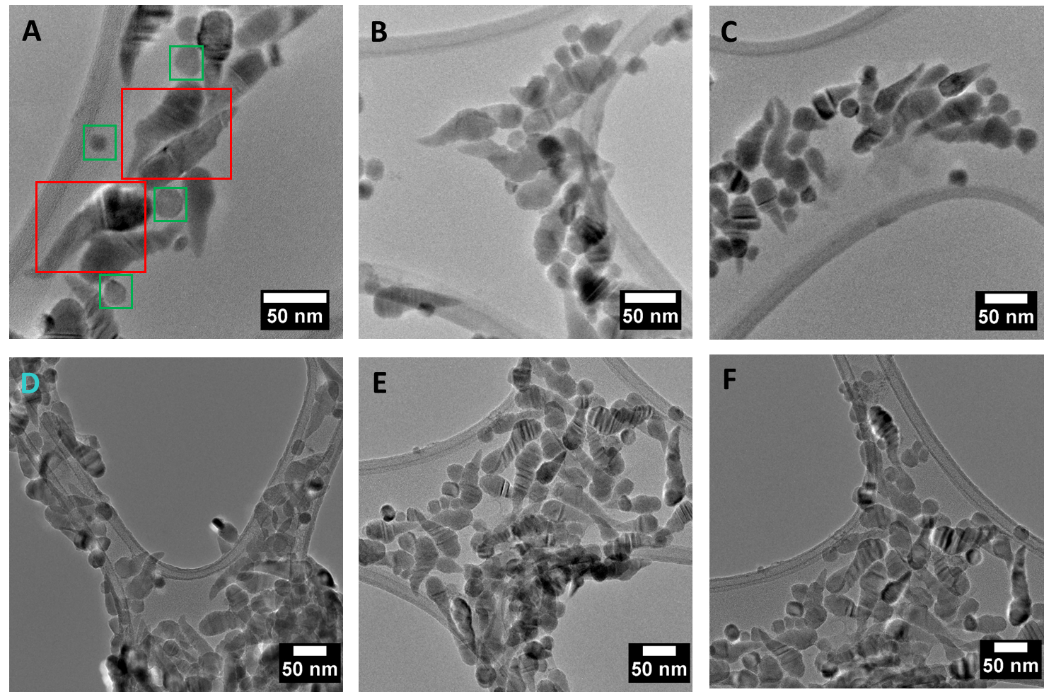


**Figure S7.** (A-F) TEM images of Cu-In-(Zn)-S/ZnS “ice-cream cone” like nanosttructures. The red and green squres in Figure S8A indicate the cone structure and the spherical particles that present in the obtianed sample.

**Table S1.** PL lifetime components for the final Cu-In-(Zn)-S/ZnS “ice-cream” like structure.

| Components | T_1_ (s) | T_2_ (s) |
| --- | --- | --- |
|  | 7.680894E-08 | 4.285024E-07 |
| Amplitude | B_1_ | B_2_ |
|  | 0.177 | 0.823 |

Excitation-Emission Matrix

Correction Factor Applied

The correction factor (C_f_) considers the absorption at excitation (A_ex_) and the emission wavelength (A_em_) , using the equation s1 to calculate the correction factor. This can be used to calculate. Using the correction factor, it is possible to correct the initial intensity (I_obs_) to the corrected intensity (I_c_) of emission spectra, using equation S2.

$${C_{f}=10}^{\frac{\left( A_{ex}+A_{em} \right)}{2}}$$

**Equation S1.** Used to calculate the correction factor

$$I_{c}=I_{obs}{\times C}_{f}$$

**Equation S2.** Used to calculate the corrected intensity from the intensity observed

**
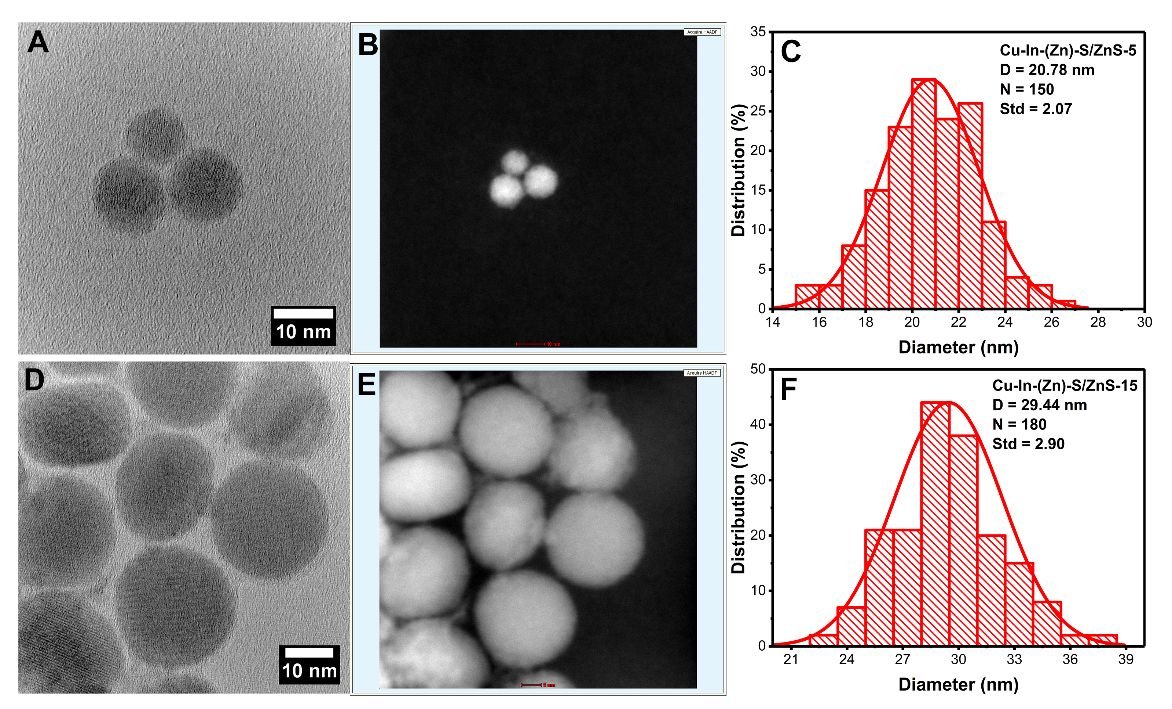
**

**Figure S8.** (A) TEM image, (B) STEM image and (C) Diameter distribution histogram of Cu-In-(Zn)-S/ZnS-5. (C) TEM image, (D) STEM image and (F) Diameter distribution histogram of Cu-In-(Zn)-S/ZnS-15.

Blank experiment

Briefly, 0.0952 g (0.5 mmol) of CuI, 0.146 g (0.5 mmol) of In(Ac)_3_, 0.6323 g (1 mmol) of Zn(St)_2_,10 mL (8 mmol) of 1-DDT, 5 mL of OAm and 10 mL of ODE were mixed in a 100-mL three-neck round-bottom flask. The mixture was degassed for 10 min at RT and 20 min at 100 $℃$. Under argon atmosphere, the temperature was increased to 230 $℃$. After 2 h, the heating mantle was removed, the reaction vessel was allowed to cool to RT. Toluene and ethanol were used to clean the sample with centrifugation. The clean sample was stored in toluene in the fridge (4 $℃$).


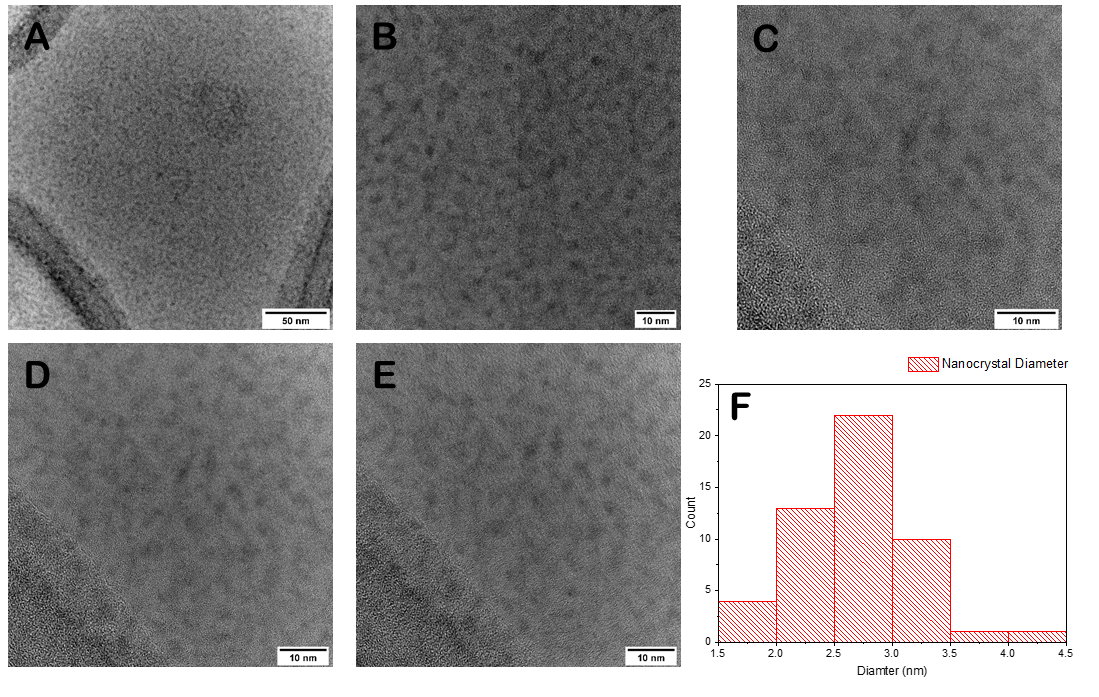


**Figure S9**. (A-E) TEM images of the obtained QDs and (F) the size histogram of the sample.

**Table S2.** The summary of the obtained Cu-In-S QDs after changing the reaction conditions.

| Sample | Reaction temperature ($℃$) | Reaction time (h) | Size of QDs (nm) |
| --- | --- | --- | --- |
| 1 | 210 | 2 | 2.7 |
| 2 | 210 | 4 | 3.2 |
| 3 | 230 | 2 | 3.3 |


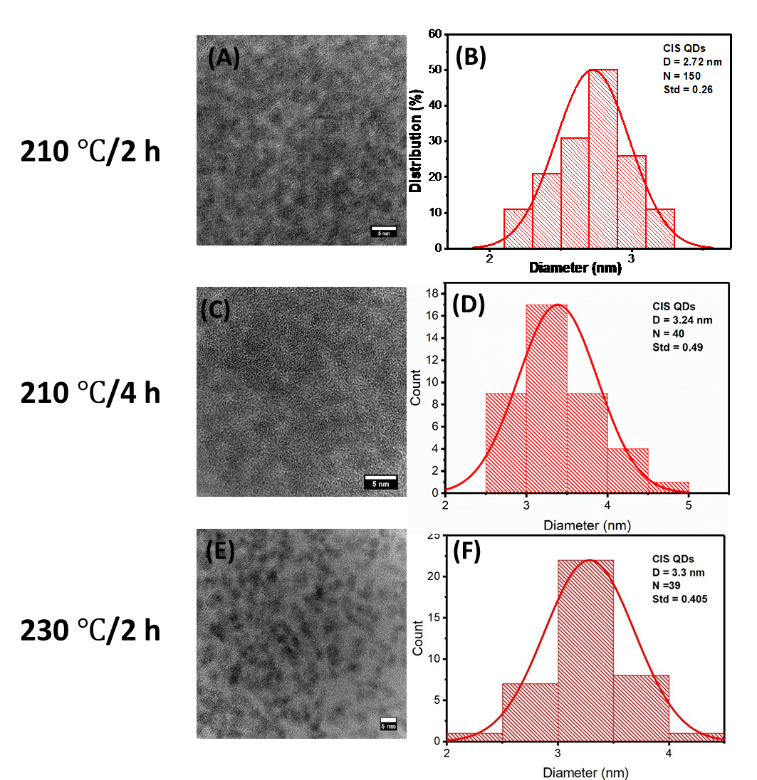


**Figure S10.** (A-B) TEM image and the size distribution histogram of the Cu-In-S QDs that prepared at 210 ℃ for 2 h, (C-D) TEM image and the size distribution histogram of the Cu-In-S QDs that prepared at 210 ℃ for 4 h and (C-D) TEM image and the size distribution histogram of the Cu-In-S QDs that prepared at 230 ℃ for 2 h.
